# Role of autophagy in antiviral innate immunity

**DOI:** 10.1186/s11658-026-00871-6

**Published:** 2026-02-24

**Authors:** Jiufeng Xie, Pengtao Jiao, Dong Wang, Manyu Shi, Cui Yuan, Lijuan Su, Guozhi Zhang, Yuhe Wang, Zhenling Ma, Liqing Li, Wei Liu

**Affiliations:** 1 College of Life Sciences, Henan Agricultural University, Zhengzhou, 450002 China; 2 Institute of Animal Science, Chinese Academy of Agricultural Sciences, Beijing, 100193 China; 3 Department of Genomic and Computational Biology, University of Massachusetts Chan Medical School, Worcester, 01605 USA; 4 No.984 Hospital, PLA Joint Logistics Support Force, Beijing, 100094 China

**Keywords:** Autophagy, Virus, Viral infection, Innate immune response, Pattern recognition receptor

## Abstract

Autophagy exerts an important effect on preserving homeostasis of cellular metabolism through degrading superfluous intracellular components. In addition, it serves as the defense mechanism for eliminating invading pathogens, such as viruses, by the host. The onset of a viral infection triggers autophagy, thereby initiating the innate immunity via the pattern recognition receptor pathways. As a result, interferons and other proinflammatory factors are produced. Furthermore, autophagy specifically targets immune components linked to viral particles for degradation. Through presenting virus-derived antigens to T lymphocytes, this process supports adaptive immunity. Nonetheless, certain viruses evolve mechanisms for inhibiting autophagy, enabling evasion of degradation and immune detection, since autophagy is frequently related to inflammatory diseases, including infections, autoimmune disorders, cancer, metabolic syndromes, neurodegenerative conditions, and cardiovascular and liver diseases. This review aims to summarize the current knowledge regarding the key molecules and specific molecular mechanisms that underlie the pattern recognition receptor signaling where autophagy is implicated during viral infections.

**Graphical abstract:**

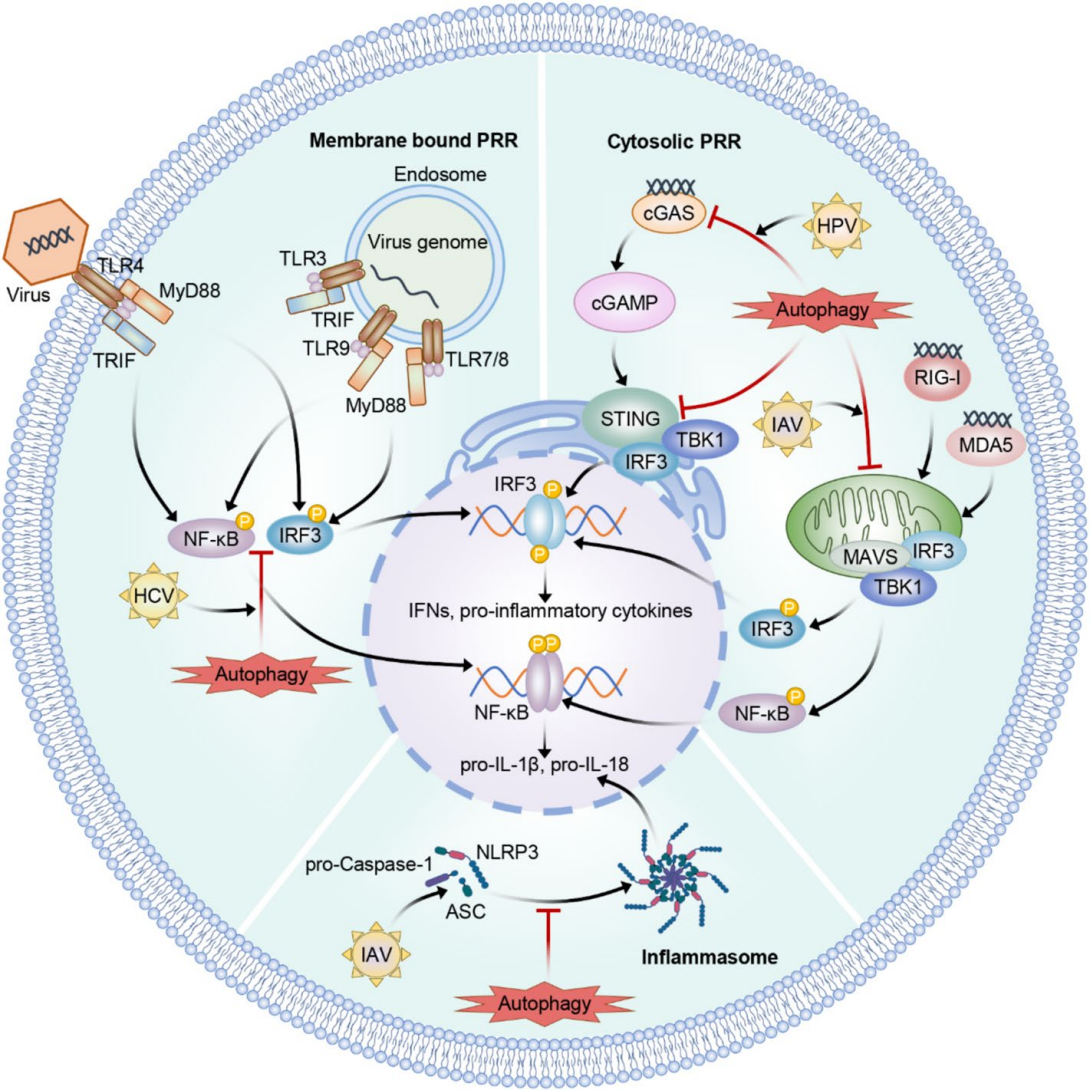

## Introduction

Autophagy can decompose unneeded intracellular contents for maintaining cellular metabolism. Besides, it is the host defense mechanism removing exogenous substances such as invading viruses [1, 2]. In mammals, it has three major activities: metabolism, defense, and quality control, and they are closely associated in immunity [3]. Autophagy can be mainly classified as two subtypes according to the degradation targets: (1) “bulk” or “nonselective” autophagy, including random organelle and cytoplasmic component sequestration and degradation; (2) “selective” autophagy, targeting specific substrates for degradation [4]. Nonselective autophagy facilitates cell survival in nutrient deprivation through maintaining metabolic processes till nutrient availability is restored [5].

Selective autophagy plays an essential role in maintaining cellular balance through promoting degradation of targeted substrates, including impaired organelles, misfolded proteins, and invading pathogens that potentially threaten cells [6, 7]. The above process can be regulated by some receptors and divided as ubiquitin-dependent or ubiquitin-independent pathways. Multiple sequestosome-like receptors (SLRs), such as p62/SQSTM1, participate in the ubiquitin-dependent pathway [8, 9]. In addition, selective autophagy is further divided on the basis of its targets, including lysophagy for lysosomes, xenophagy for intracellular pathogens, ER-phagy for the endoplasmic reticulum (ER), mitophagy for mitochondria, pexophagy for peroxisomes, aggrephagy for protein aggregates, ribophagy for ribosomes, glycophagy for glycogen, ferritinophagy for ferritin, lipophagy for lipid droplets, as well as fluidophagy for fluid droplets, etc. [6, 10, 11]. In selective autophagy, target cargos are associated with different physiological functions. The failure in degrading such cargos often leads to pathogenic mechanisms in human conditions such as cancer, metabolic issues, dysregulated inflammation, neurodegenerative diseases, and infectious diseases [12–14].

In autophagy, excess and defective cytosolic components or organelles will be secluded inside the double-membrane vesicle, and it subsequently merges with vacuole or lysosome to form an autophagolysosome [15]. Within this structure, the encapsulated contents are degraded by hydrolases [16]. Innate immunity is the early antiviral infection defense mechanism, and autophagy is crucial for regulating various innate immune pathways [17]. During viral infection, pattern recognition receptors (PRRs), including nod-like receptors (NLRs), Toll-like receptors (TLRs), retinoic-acid-inducible gene I (RIG-I)-like receptors (RLRs), and cyclic GMP–AMP (cGAMP) synthase (cGAS), identify pathogen-associated molecular patterns (PAMPs) while activating the downstream signaling cascade. This activation leads to the production of type I interferons (IFN-I) and cytokines, thereby enhancing innate antiviral immunity [18]. Subsequently, IFN-I initiates interferon-stimulated gene (ISG) transcription, as a result, proteins with antiviral functions are produced [19]. Besides, autophagy exerts the fundamental effects on removing cytoplasmic components; consequently, it is triggered by innate immunity to degrade and remove invading viral particles. In the later stages of infection, autophagy accelerates antigen processing and activates adaptive immunity [20].

Virus-induced autophagic degradation, first called virophagy, is referred to as xenophagy, and represents an important antiviral defense mechanism. It targets viral proteins and viruses for degradation, and promotes host immunity, such as regulating inflammation while recognizing and presenting antigens [21–24]. Nonetheless, some viruses evolve mechanisms for evading autophagy and thus avoid immune detection and degradation [2, 16, 25]. Besides, certain viruses may cause autophagy and later use autophagosomes to be replication sites or promote viral particle maturation and release through manipulating autophagic secretion pathway, finally accelerating viral replication and transmission [26, 27]. Many viruses use different strategies for circumventing diverse selective autophagy types, so as to manipulate the above processes for modulating immune responses and organelle degradation [28–30]. Recently, molecular interactions between innate immune pathways and virus-induced autophagy are illustrated, suggesting that viruses develop different strategies to inhibit or exploit autophagy, finally promoting viral replication and survival. In this review, we present a comprehensive analysis of the physiological significance and molecular mechanisms of autophagy in regulating antiviral innate immunity upon viral infections. Our aim is to underscore the therapeutic potential of regulating autophagy to enhance antiviral responses and to highlight promising avenues for the development of effective treatments against viral infections.

## Autophagy: process and regulation

**Fig. 1:**
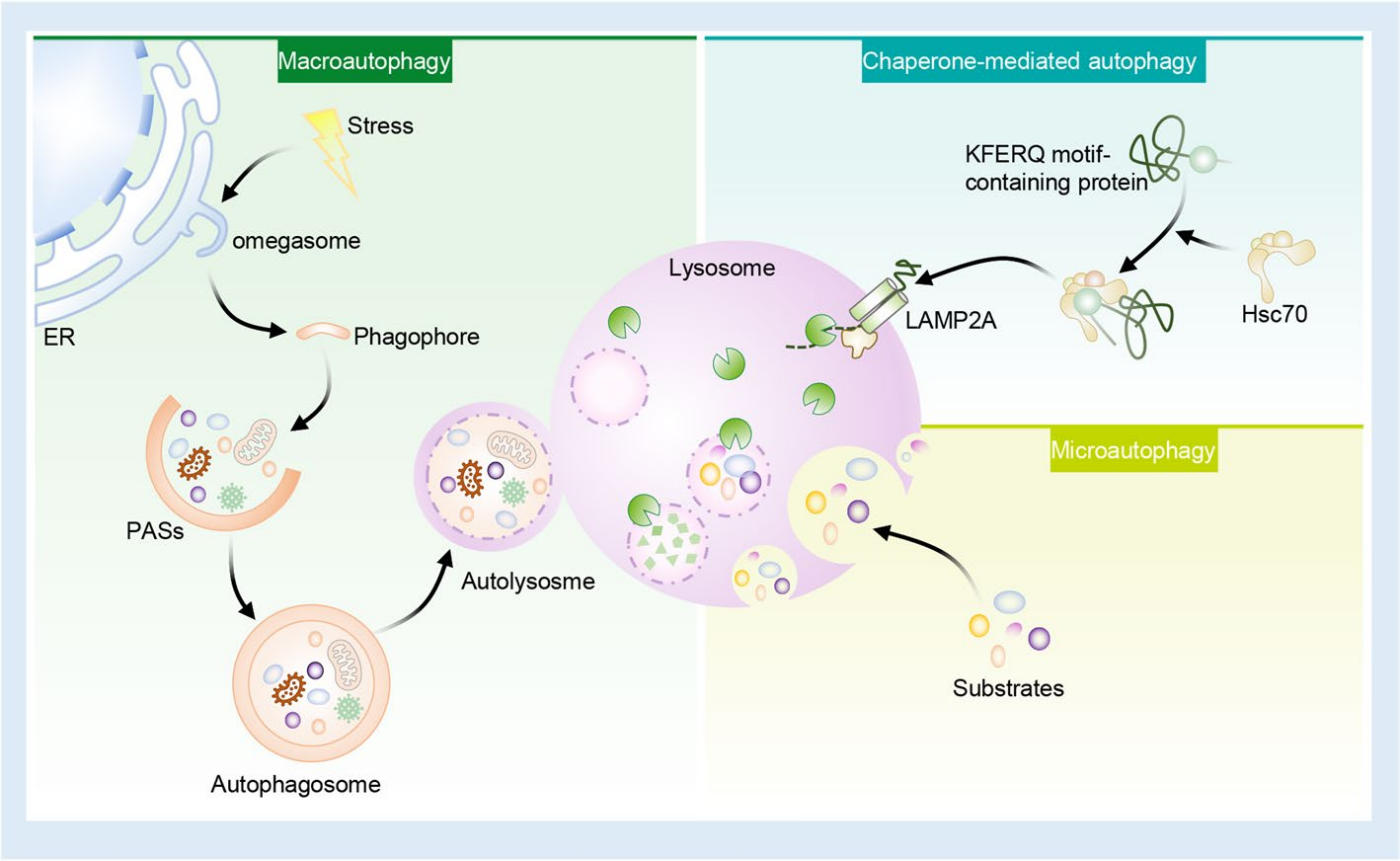
The types of autophagy. There are three types of autophagy: chaperone-mediated autophagy (CMA), microautophagy, and macroautophagy. In CMA, KEFRQ motif in target proteins can be recognized by and combined with chaperone protein HSC70. Such complexes will then be delivered to lysosome by interacting with lysosomal receptor LAMP2A. Microautophagy is related to sequestrating cytosolic components by lysosomal membrane invagination, as well as separation and degradation inside lysosome. Macroautophagy is characterized by double-membrane vesicle (called autophagosome) generation, finally fusing with lysosome for facilitating degradation

Autophagy is categorized in microautophagy, chaperone-mediated autophagy (CMA), or macroautophagy according to cargo delivering mechanisms to lysosomes. Microautophagy witnesses the invagination of lysosomal membrane for directly engulfing cytosolic components, forming vesicles within the lysosome [15]. CMA involves the selective autophagic recognition of the KFERQ motif on target proteins via intracellular chaperone Hsc70 (heat shock cognate protein, 70 kDa), facilitating a lysosomal-associated membrane protein 2A (LAMP2A)-induced transfer to lysosomes where it is degraded [31]. Macroautophagy differs from the other two forms due to autophagosome, the special double-membrane structure enclosing cytosolic components for fusion with the lysosome, which facilitates the breakdown of the cargo [32]. Autophagy, also known as macroautophagy, has been extensively investigated and shows a high conservation degree across species ranging from yeast to mammals (Fig. 1).

**Fig. 2:**
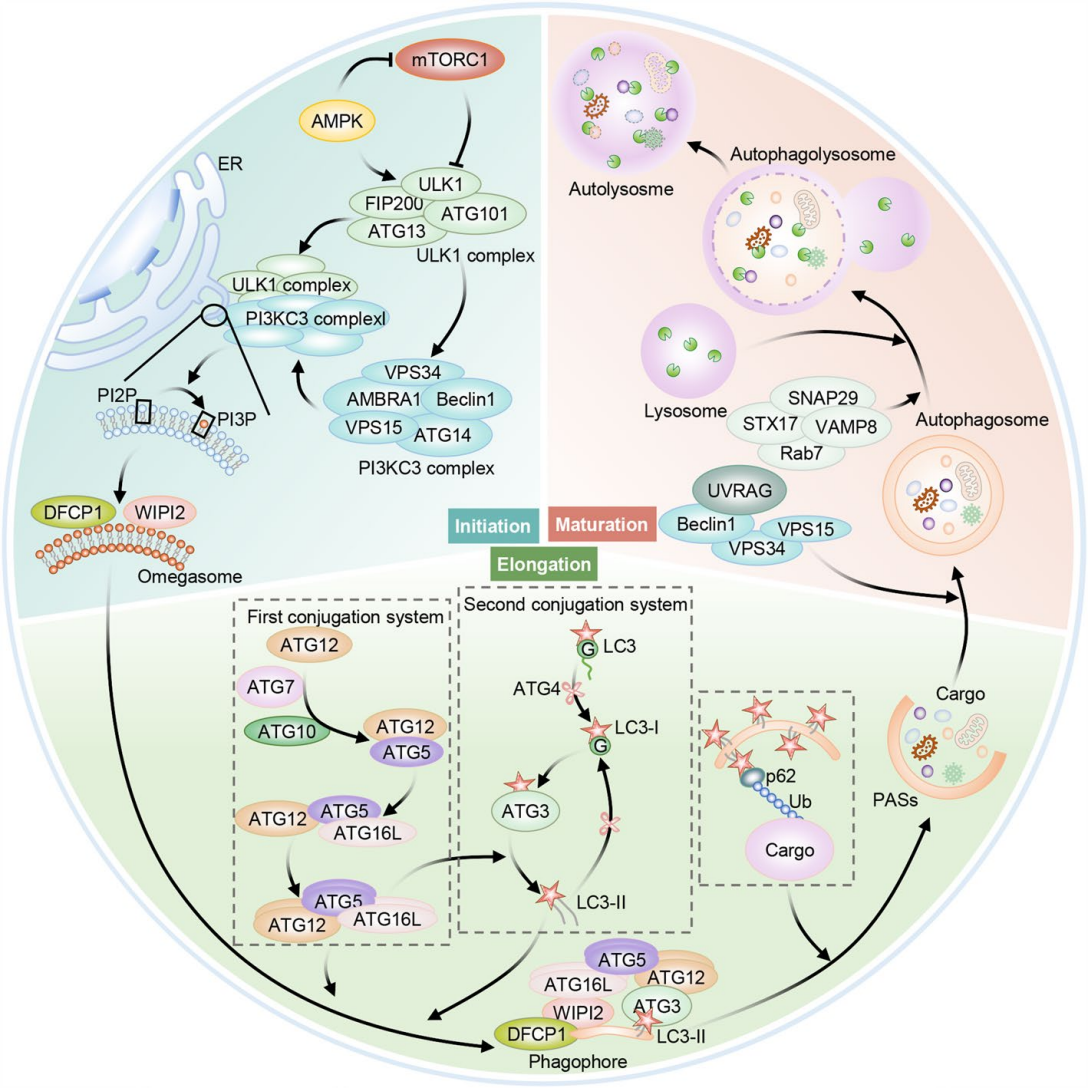
Canonical autophagy includes initiation, elongation, and maturation stages. Its initiation stage is under the physiological regulation by mTORC1’s repression effect, promoting inactivation of ATG13 and ULK1 phosphorylation. The mTORC1-induced inhibition can be reversed by AMPK, and this can phosphorylate Beclin-1 and ULK1 when ATP levels are reduced. ULK1 enhances autophagy through promoting PI3K activity of the multiprotein complex consisting of PIK3R4–VPS15, PIK3C3–VPS34, Beclin-1, and ATG14. Phagophore expansion promotes the elongation phase, which involves two different Ub-like conjugation systems. The first one is dependent on ATG7 and ATG10 activities, which enables the assembly of the multiprotein complex containing ATG5, ATG12, alongside ATG16L1. The second system is associated with ATG3 and ATG4 that can cleave ATG8-family proteins, including mammalian MAP1LC3/LC3. The cytosolic LC3 protein, with diffuse expression, is conjugated to phosphatidylethanolamine (PE) in this process, which then enables its integration in expanding phagophore membrane as LC3-II. LC3-II is the receptor for LC3-interacting regions (LIR)-proteins, such as autophagy receptors and substrates including SQSTM1/p62. After the phagophore is closed, the resulting autophagosome fuses with lysosome, forming an autolysosome, finally resulting in autophagic substrate degradation via acidic lysosomal hydrolases

Autophagy induction is initiated by phagophore formation, a process controlled via autophagy-related (ATG) proteins drawn onto various organelle membranes for initiating autophagy [33]. The phagophore encloses impaired organelles, protein aggregates, or misfolded proteins, leading to nucleation and creation of preautophagosomal structures (PASs) that extend into autophagosomes by the recruitment of additional membrane structures [34]. Then, autolysosomes are formed through autophagosomes fusion with lysosomes, where those captured substances will be decomposed via lysosomal proteases [35]. Thus, autophagy involves three main sequential stages: initiation, elongation, and maturation (Fig. 2).

The initiation process entails activation of Unc-51-like kinase 1 (ULK1)-ATG13-focal adhesion kinase family interacting protein of 200 kD (FIP200, called RB1CC1 as well) kinase complex, mediated by signals from adenosine monophosphate (AMP)-activated protein kinase (AMPK) and mammalian target of rapamycin (mTOR) complex 1 (mTORC1) [36, 37]. mTORC1 inactivates autophagy by interaction with ATG1/ULK1 complex, which comprises ATG1, ATG13, ATG101, ULK1, and FIP200, and phosphorylation of ULK1 and ATG13 [38]. Inhibition of mTORC1 results in autophagy activation, facilitating mTOR substrate complex translocation from the cytosol into specific ER domains or adjacent structures [39]. Such translocation enhances the class III phosphatidylinositol-3-OH kinase (PI3K) complex assembly, comprising VPS15, VPS34, ATG14, and Beclin-1, to the ER [40]. Two distinct complexes are formed by Beclin-1 and VPS34: complex I, associated by ATG14L, and complex II, associated by UVRAG. Functionally, the Beclin-1–VPS34 complex I promotes autophagosome generation, whereas the latter controls vacuolar protein-sorting pathway [41]. PAS formation is aided by PI3KC3 through producing phosphatidylinositol-3-phosphate (PtdIns(3)P), while the latter lures effectors such as DFCP1 (double FYVE-containing protein 1) and WIPI (WD-repeat domain phosphoinositide-interacting) family proteins [42]. DFCP1 is capable of translocating from the endoplasmic reticulum (ER) or Golgi to autophagosome site via the PtdIns(3)P-dependent manner for forming omegasomes, the ER-associated Ω-like structures [43]. The autophagic vesicle membrane, which originates from the mitochondria, ER, or plasma membrane, is formed with the assistance of initiation and nucleation proteins [44].

The elongation phase involves phagophore expansion through two intricate ubiquitination (Ub)-like conjugation complexes. The first conjugation system enables ATG5–ATG12 conjugation through mediating E1-like ubiquitin-activating enzyme ATG7 as well as E2-like ubiquitin-conjugating enzyme ATG10 [45, 46]. Later, ATG16L1 noncovalently associates with ATG5–ATG12 conjugates [47, 48]. In a parallel conjugation system, ATG7 and E2-like ubiquitin-conjugating enzyme ATG3 facilitate ATG5–ATG12–ATG16L1 complex assembly, and functions analogously to an E3 ubiquitin ligases in conjugating ATG8 to the lipid phosphatidylethanolamine (PE) [49]. Thereafter, E1 and E2 enzymes, ATG7 and ATG3, mediate a series of ubiquitylations, resulting in ATG12 and microtubule-associated protein 1 light chain 3 (MAP1LC3/LC3) forming ATG12–ATG5–ATG16L complex and lipidated LC3-II, separately [50]. By anchoring to the PAS membrane, these products aid in its extension by recruiting additional membrane components. Ultimately, autophagosomes are formed, encapsulating their substrates [51]. Numerous cargo receptors, including SQSTM1/p62, detect ubiquitination signals through their ubiquitin-associated (UBA) domain and interact with LC3 family proteins using their LC3-interacting region that is located in phagophore membrane. This interaction facilitates autophagosome formation, leading to the degradation of cargo [52].

In the maturation stage, the autophagosome merges with a lysosomal compartment through a process facilitated via UV resistance-associated gene (UVRAG)-containing class III phosphatidylinositol 3-kinase (PtdIns3K) complex [53]. This process also involves several other components, including Rab7 GTPase and soluble N-ethylmaleimide-sensitive factor attachment protein receptor (SNARE) complex, which comprises synaptosomal-associated protein 29 (SNAP29), syntaxin 17 (STX17) as well as vesicle-associated membrane protein 8 (VAMP8) [54]. After autophagosomes merge with lysosomes, an autolysosome, which is a structure where lysosomal enzymes degrade the trapped materials, is created. When additional autophagosomes fuse with lysosomes, the autolysosome size increases. In the termination phase, lysosomes undergo tubulation and fragmentation to facilitate their renewal [55].

## Autophagy-mediated antiviral innate immunity

**Fig. 3:**
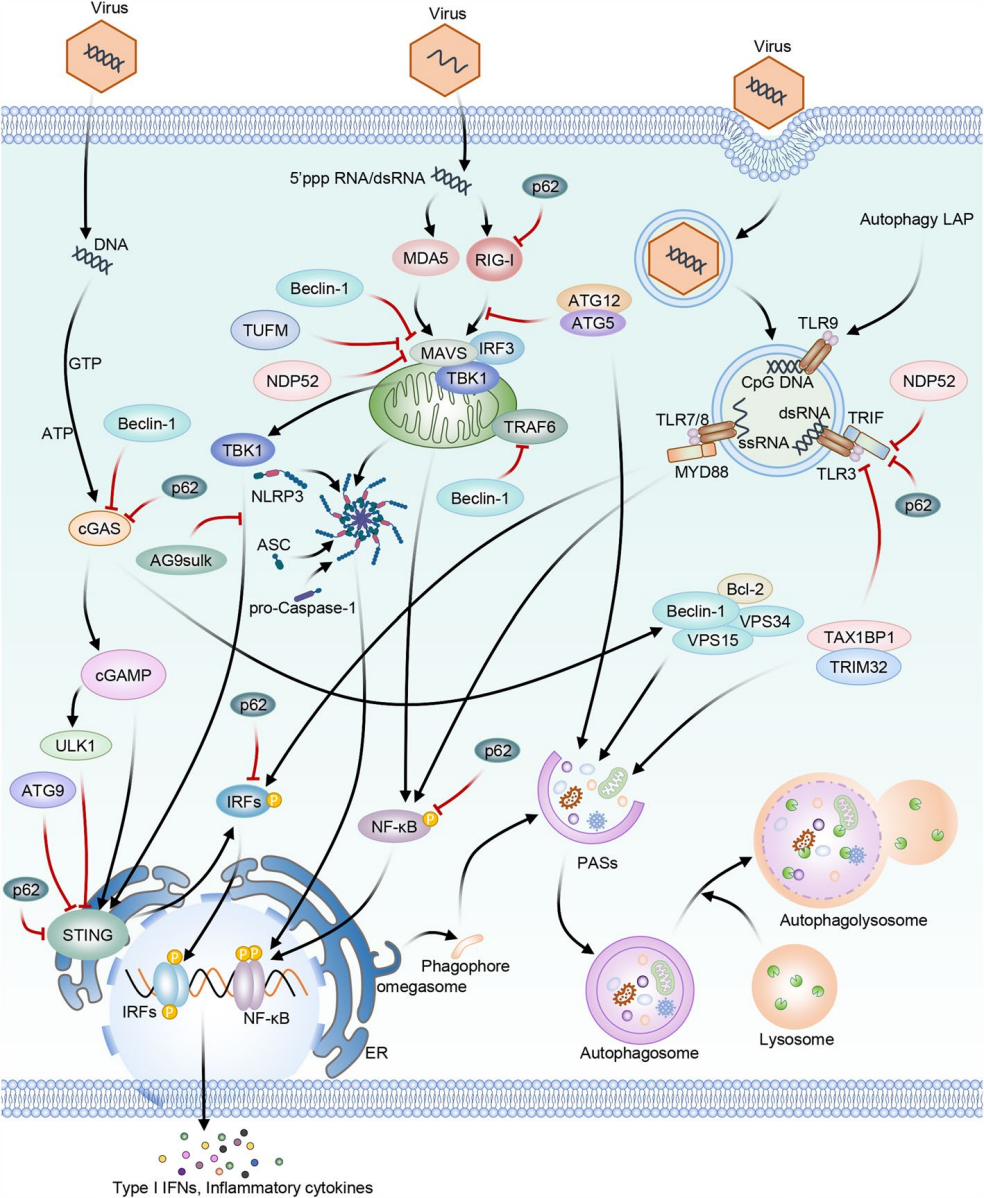
Autophagy modulates immune sensing pathways postviral infection. After infection, specific PRRs can be activated according to specific PAMPs. Autophagy can promote PAMP recognition to enhance innate immunity, thus increasing cytokine production and regulating the immunity for mitigating side reactions related to prolonged immune activation. Solid and T-shaped arrows represent stimulation and inhibition separately

Autophagy has a key effect on innate immunity as it facilitates to regulate various innate immune pathways. It is kept at the basic level under physiological conditions, but upregulated upon diverse stimuli, including energy shortage, nutrient deprivation, damaged organelles, misfolded proteins, inflammation, and infections [53, 56–58]. During viral infections, PRRs such as RLRs, TLRs, cGAS, and NLRs activate autophagy. This activation enhances the innate immune response by upregulating IFN-I, inflammatory cytokines, chemokines, and various antipathogenic genes [59]. However, dysregulated innate immunity activation may lead to inflammatory disorders, including septic shock and autoimmune disorders. Consequently, the host possesses a sophisticated regulatory system for innate immunity for preventing excess or insufficient immune responses. Nonetheless, interactions of autophagy with primary innate signaling pathways activated throughout viral infections (Fig. 3) probably promote occurrence of infectious disorders and inflammatory conditions.

### RLR signaling pathway regulated by autophagy

RLRs detect foreign cytosolic RNA using primary sensors RIG-I, laboratory of genetics and physiology 2 (LGP2), or melanoma differentiation-associated protein 5 (MDA5) [60]. Such sensors have the ATP-dependent RNA helicase domain to recognize double-stranded RNA (dsRNA), as well as the C-terminal repressor domain (CTD) for binding RNA [61]. RIG-I targets single-stranded RNA (ssRNA) or dsRNA with the 5′ diphosphate or triphosphate, which is typical of ssRNA viruses, while MDA5 detects long dsRNA, crucial for both ssRNA and dsRNA viral infections [62]. LGP2, which lacks N-terminal caspase activation and recruitment domains (CARD), modulates the MDA5 and RIG-I functions [63]. Upon detecting dsRNA, the RLRs experience conformational alterations and oligomerization, exposing CARDs for recruiting the signaling adaptor called mitochondrial antiviral-signaling (MAVS) localized on mitochondrial outer membrane through C-terminal transmembrane (TM) domain [64, 65]. Upon activation, it forms a prion-like structure that facilitates the assembly of a signalosome, activating kappa B kinase ε (IKKε) and TANK binding kinase 1 (TBK1) [66]. These kinases are capable of phosphorylating interferon regulatory factor 3 (IRF3) and nuclear factor kappa B (NF-κB), accelerating their nuclear translocation as well as transcription of inflammatory factor and IFN-I [67]. Moreover, MAVS exists on peroxisomes, in which it activates IRF1 through binding to RIG-I, thereby generating ISGs or type III IFN [68]. Such RLR-induced IFN generation promotes host immunity to eliminate viruses and monitor cancer.

The role of autophagy in negatively regulating RLR-related antiviral signaling has been increasingly reported [69]. Certain viruses utilize autophagy for degrading organelles and molecules related to RLR-induced IFN response. The ATG5–ATG12 complex, functioning independently from autophagy, inhibits RIG-I–MAVS interaction by binding to CARD domains, finally suppressing their activation [70]. Likewise, as the glucose-based molecule, trehalose facilitates autophagy and promotes ATG5 and RIG-I–MAVS interaction, decreasing intracellular IFN-λ generation after human rhinovirus 16 (HRV-16) infection [71]. In addition, Beclin-1 blocks RIG-I–MAVS interaction through its interaction with MAVS CARD, inhibiting RIG-I signal transduction [72]. Ke et al. reported that hepatitis C virus (HCV)-mediated autophagy inhibits RIG-I signal transduction and IFN-I generation, similar to the effects observed during Dengue virus (DENV) infection [73]. Although the exact mechanisms remain unclear, HCV-induced autophagy increases ATG5 and Beclin-1 expression, inhibiting IFN-I generation by negatively affecting RIG-I signaling. RIG-I acts as the degradative substrate for autophagy that results from RNA viruses such as vesicular stomatitis virus (VSV) and H1N1 influenza A virus (IAV). As discovered by Du et al., leucine-rich repeat containing protein 25 (LRRC25), a novel regulator, is interactive with ISG15-modified RIG-I for promoting the interaction with autophagic receptor p62, causing RIG-I selective autophagy and decreased IFN-I generation [74]. However, Thinwa et al. discovered that the kinase CDKL5 triggers virus-induced autophagy, protecting neurons and mice from mortality during the virophagy associated with Sindbis virus (SINV) infection. Specifically, CDKL5 phosphorylates p62 at T269/S272 residues, allowing p62 to capture viral capsid aggregates, which is vital for selective autophagy and defense against neurotropic viruses [23].

Furthermore, autophagy has the potential to block RLR-induced IFN-I signal transduction through aiming at MAVS. ATG5 deficiency obstructs autophagy, as a result, defective mitochondria and mitochondrial reactive oxygen species (ROS) accumulate, thereby amplifying RLR-mediated IFN-I signal transduction [75]. Therefore, eliminating impaired mitochondria through autophagy may be important for keeping balance of the signaling. MAVS is localized in mitochondria, suggesting that autophagy is vital for mitochondrial activation and dynamics. Mitofusin, a mitochondrial fusion protein, exerts an essential effect on IFN-I generation and MAVS aggregation [76]. As reported by Ding et al., human parainfluenza virus type 3 (HPIV3) triggered mitophagy to subsequently decompose MAVS and disrupt RIG-I-mediated IFN-I responses [77]. During infections with Japanese encephalitis virus (JEV), suppressing cell autophagy promotes MAVS aggregation, thereby increasing proinflammatory factor contents [78]. Throughout IAV infection, PB1-F2 protein does not interact with Tu translation elongation factor mitochondrial (TUFM), which thus induces mitochondrial fragmentation and mitophagy to decompose MAVS and inhibit IFN-I generation, eventually impairing innate immunity [79]. Likewise, the severe acute respiratory syndrome coronavirus (SARS-CoV-2) impairs innate immunity with M and ORF10 proteins, thereby inducing MAVS degradation, an important mitochondria-related antiviral component, eventually reducing IFN-I generation [80, 81].

As illustrated by Xie et al., NS5A protein within classical swine fever virus (CSFV) promoted Beclin-1–MAVS interaction to reduce IFN-I level [82]. The more severe autophagy inhibits CSFV-induced JAK–STAT and RIG-I–IRF3 pathways to suppress IFN-I generation. Therefore, the above process decreases NF-κB pathway inhibition and apoptosis of CSFV-infected cells [83]. Tetherin also recruits E3 ubiquitin ligase MARCH8 to generate the K27-linked ubiquitin chains on MAVS, which then triggers NDP52-induced autophagic degradation [84]. Further, RING finger protein 34 (RNF34) promotes MAVS ubiquitination through NDP52 to achieve autophagic degradation [85]. In addition to targeting VSV and SeV, NDP52/Calcoco2 degrades MAVS to suppress RLR signaling pathway in Coxsackievirus B3 (CVB3)-infected cells [86]. Furthermore, CVB3 3C protease (3Cpro) cleaves SNAP29 and PLEKHM1, suppressing autophagosome–lysosome fusion while enhancing autophagosome accumulation to facilitate viral replication [87].

IRF serves as an important regulatory target and signaling molecule. As reported by Kim et al., Rubicon (the autophagy inhibitor) interacted with IRF, reduced IFN-I generation and inhibited IRF3 dimerization to suppress RLR signaling alongside antiviral responses [88]. Moreover, viral stimulation upregulates IFN-induced transmembrane protein 3 (IFITM3), thereby enhancing IRF3 autophagic degradation and regulating antiviral responses by means of negative feedback [89]. Xie et al. reported that the deubiquitinase OTUD7B exerts the negative regulatory effect through deubiquitinating SQSTM1/p62, thereby enhancing its activity to degrade IRF3 [90]. Furthermore, RIG-I activation induces an association between Beclin-1 and the tumor necrosis factor receptor-associated factor 6 (TRAF6) on mitochondria, resulting in TRAF6 K63-linked polyubiquitination and initiating autophagy [91].

### TLR signaling pathway regulated by autophagy

TLRs are crucial for activating immune cells and defending against microorganisms. These transmembrane proteins comprise extracellular leucine-rich repeat (LRR) motifs to recognize ligands and the cytoplasmic Toll/interleukin 1 (IL-1) receptor (TIR) domain for signal transduction [92]. According to the cellular location, TLRs are divided as two subfamilies: plasma membrane TLRs (such as TLR1, TLR2, TLR4, TLR5, TLR6, TLR10) and endosomal TLRs (such as TLR3, TLR7, TLR8, TLR9, TLR11, TLR12, TLR13), with the latter primarily involved in antiviral responses [93, 94]. Each TLR specifically interacts with PAMPs, and these interactions are strengthened by TLR homo- or heterodimer generation. They mainly act against viral infection during immune responses [95]. For instance, TLR3 detects dsRNA, TLR7, and TLR8 can detect ssRNA, whereas TLR9 can detect unmethylated CpG DNA, and TLR2 and TLR4 recognize viral proteins, thereby contributing to antiviral immunity [96–98]. Upon engagement with their respective agonists, TLRs recruit adaptor molecules including myeloid differentiation factor 88 (MyD88), Toll-receptor-associated activator of interferon (TRIF), Toll-receptor-associated molecule (TRAM), MyD88-adaptor-like/TIR-associated protein (MAL/TIRAP), and Sterile α- and armadillo-motif containing protein (SARM), to transmit TIR signals, which then activate kinases and transcription factors such as NF-κB, IRF, and mitogen-activated protein kinases (MAPKs) [99, 100]. Such signaling cascade ultimately produce IFN-I and inflammatory cytokines, which mediate immune responses to PAMPs [101].

Autophagy is important for regulating and triggering TLR/NF-κB pathway, with TLR7 ligands being strong autophagy inducers. TLR7 detects viral RNA, aiding autophagy in transporting viral intermediates into lysosomes while producing IFN-α within plasmacytoid dendritic cells (pDCs) [102]. In human immunodeficiency virus (HIV)-1 and aramyxovirus simian virus 5 (SV5) infections, autophagy activates TLR7 in pDCs, leading to antiviral cytokine production [103, 104]. In HCV infections, TLR7 and TLR8 detect HCV RNA, promoting tumor necrosis factor-alpha (TNF-α) production [105]. Nonetheless, HCV-related autophagy decreases TNF receptor-associated factor 6 (TRAF6) expression to limit inflammatory factor generation [106]. Noncanonical autophagy, in particular LC3-associated phagocytosis (LAP), affects TLR9 activation in different cells [107]. TLR9 trafficking and its activity are dependent on autophagy-related proteins, and these two processes occur within murine pDCs treated with DNA-immunoglobulin complexes in a ULK1-independent manner [108]. According to Severa et al., autophagy induced TLR signaling in pDCs infected with Epstein–Barr virus (EBV) [109]. TLR9 activation within macrophages via CpG dinucleotides contributes to recruiting IKK-β and LC3B to TLR9-positive endosomes, thereby upregulating IFN-I level [110]. After IAV exposure, autophagy helps localize H1N1 IAV inside lysosomes, activate TLR signaling, and promote T-helper cell differentiation within bone marrow-derived dendritic cells (BMDCs) [111]. Therefore, TLR signaling is closely associated with autophagy.

Autophagy can negatively affect such pathways through targeting various TLR-associated factors for degradation [112]. For instance, in bronchial epithelial cells infected with coxsackievirus A16 (CA16) and enterovirus 71 (EV71), autophagy decreased TLR7 activation and IFN-I production [113]. Selective autophagy also modulates TLR signal transduction; in polyinosinic-polycytidylic acid (Poly(I:C)) treatment, the autophagic receptor NDP52 inactivates IRF3 through degrading adaptor proteins TRIF and TRAF [114]. Samie et al. reported that TRIF filaments generated when TLR is activated are degraded via p62/SQSTM1- and TAX1BP1-induced autophagy [115]. In addition, within macrophages treated with lipopolysaccharide (LPS) or Poly(I:C), TRIM32 targets TRIF through TAX1BP1-mediated autophagy to suppress TLR3/4-mediated IFN-I responses [116, 117]. Furthermore, the NF-κB signaling pathway components interact with autophagic processes. IKK-β undergoes selective autophagy via p62/SQSTM1, influenced by SKP2 [118], which is affected by TRIM21-mediated ubiquitination [119] or phosphorylation inhibition [120]. The NF-κB subunit p65/RelA is degraded lysosomally, with LRRC25 and p62/SQSTM1 facilitating its autophagic degradation [121]. In contrast, the KFERQ motif in p65/RelA enables its binding to Hsc70, which results in CMA-mediated degradation [122]. Autophagy also affects noncanonical NF-κB pathway, because prolonged TNF-α exposure inside bone marrow-derived macrophages (BMDM) targets p62/SQSTM1 to NF-κB p100/p52 dimers for autophagic degradation [123].

In viral infections, the PRRs-autophagy interaction adjusts intracellular signaling to create an optimal antiviral state [124]. Although some viruses trigger autophagy to avoid immune defenses, autophagy primarily functions to enhance viral recognition via TLR signaling. This represents an antiviral strategy that operates independently of degradation processes. In addition, upregulating autophagy in innate immunity is the potential antiviral infection target. Consequently, the TLRs-autophagy interaction promotes the viral invasion response efficacy of both systems.

### NLRs signaling pathway regulated by autophagy

The NLR protein family refers to PRR responsible for initiating innate immunity to resist cell stress and injury [125]. Such proteins have similar structures, encompassing central nucleotide-binding domains (NBDs) and C-terminal LRR domains in the NACHT domain [126]. NLRs are categorized into subfamilies on the basis of N-terminal heterogeneities, among which, NLRC and NLRP are dominant. NLRC proteins possess CARD domains, whereas NLRP proteins contain pyrin domains (PYD). There are 14 members in the NLRP subfamily that has been extensively investigated for the effect on generating “inflammasome” upon PAMP stimulation [127]. After NLRPs are activated, they interact with Caspase-1 through engaging apoptosis-associated speck-like protein containing a CARD (ASC) adapter protein, thereby releasing proinflammatory factors including pro-IL-1β and pro-IL-18 [128]. Inflammasomes exert a key function in “pyroptosis,” a cell death pattern characterized by cytosolic component release following plasma membrane rupture, resulting in locoregional inflammation [129]. Through viral infection, inflammasomes are activated upon microbial-associated molecular pattern (MAMP, such as viral RNA and DAN) and DAMP (generated upon infection) stimulation [130].

Autophagy modulates inflammation by regulating inflammasome activation, which is important for avoiding inflammatory disorders. According to research on mouse models, such as Crohn’s disease, uveitis and sepsis models, suppressing autophagy can promote inflammation and inflammasome activation [131, 132]. To be specific, NLRP3 inflammasome can interact with autophagic processes, with its activity increasing after suppressing autophagy [133]. Such relation generates the negative-feedback loop, which assists in mitigating excess inflammatory responses via autophagic inflammasome component elimination and post-translational modifications [134]. The initial strategy is related to autophagy that directly target inflammasome. Inflammasome activation through poly(dA:dT) within macrophages causes autophagy, and creates the negative feedback loop by instructing poly-ubiquitinated ASC for degradation within lysosome [135]. IRGM1, associated with Crohn’s disease, binds to ASC and NLRP3 to promote autophagic degradation and prevent oligomerization [134]. TRIM proteins, part of innate immunity, have an essential effect on the above process. The TRIM11-AIM2 inflammasome interaction in macrophages results in selective degradation mediated by p62/SQSTM1 [136]. TRIM20 is interactive with NLRP3 inflammasome components, such as NLRP3, ASC, caspase-1, and pro-IL-1β, and modulates their activity [137]. TRIM16 functions as a specialized receptor facilitating the unconventional secretion of IL-1β. Upon lysosomal damage, the cytosolic secretory autophagy cargo, IL-1β, is identified by the specialized secretory autophagy cargo receptor TRIM16, which subsequently recruits the cargo to the LC3-II sequestration membranes [138]. Furthermore, research by Claude-Taupin et al. demonstrated that TRIM16 recruits its secretory autophagy cargo, such as mature IL-1β, along with ATG proteins involved in phagophore formation. Consequently, TRIM16 serves as a critical link between the autophagy machinery and the recognition of cytosolic IL-1β in response to lysosomal damage [139].

In an influenza virus model, infection with IAV activates NLRP3 inflammasome, increasing IL-18 and IL-1β levels [140]. According to Wang et al., IAV M2 protein promotes ROS-dependent MAVS aggregation. By competing with ATG5 and LC3B for MAVS binding, IAV M2 protein hinders autophagy, leading to decreased LC3B–MAVS and ATG5–MAVS complex generation and inhibiting MAVS aggregate degradation [141]. In addition, the IAV virulence protein PB1-F2 forms high molecular weight aggregates that are moved to inner mitochondrial membrane via outer mitochondrial membrane 40 (TOMM40) channel. This translocation decreases membrane potential and induces mitochondrial fragmentation, thereby activating NLRP3 [142]. PB1-F2 may also act as an autophagy receptor, which interacts with mitochondrial protein TUFM and LC3B to achieve complete mitochondrial autophagy. Such interaction promotes MAVS degradation while attenuating IFN-I generation [80, 143]. In addition, PB1 protein in IAV inhibits immunity through targeting MAVS to attain NBR1-induced selective autophagic degradation [144].

Secondly, autophagy aids in selectively degrading DAMPs, which reduces inflammasome activation and proinflammatory factor generation, primarily via the process of mitophagy [145]. Damaged mitochondria trigger inflammation by releasing ROS and mitochondrial DNA (mtDNA) in cytoplasm. After autophagy impairment, impaired mitochondria accumulate, which enhances inflammasome activation alongside subsequent proinflammatory factor generation [146]. During SARS-CoV-2, the accessory protein ORF10 promotes mitophagy to degrade MAVS, reducing the IFN-I response and cytokine production. Activating mitophagy reduces ROS and mtDNA release, thereby preventing cell pyroptosis driven by NLRP3 inflammasome and inflammatory factor release [80]. Similarly, IAV PB1-F2 protein induces mitophagy through the interaction of TUFM and ATG5–ATG12, leading to MAVS degradation and lower IFN-I production [79]. As suggested in a study on a sepsis mouse model, PINK1/PARKIN-induced mitophagy is important for inflammasome activation, with reduced PINK1/PARKIN levels leading to damaged mitochondria accumulation and increased inflammasome activity [147].

Furthermore, other NLRs are implicated in interactions with autophagic mechanism. Specifically, NLRX1, along with the interacting partner mitochondrial TUFM connecting ATG16L1 and ATG5–ATG12 complexes, is important for promoting autophagy [148]. Furthermore, Beclin-1 participates in interactions with NLRC4, NLRP3, NLRP4, and NLRP10 [149]. Notably, throughout phagocytosis in group A streptococci, NLRP4 is recruited onto the plasma membrane, resulting in its temporary dissociation from Beclin-1 and thereby facilitating to initiate Beclin-1-mediated autophagic responses [149]. Overall, NLRs appear to congregate autophagy-related factors in close proximity to invading microorganisms or resident mitochondria, thereby triggering autophagy activation.

### cGAS/STING signaling pathway regulated by autophagy

STING is crucial for initiating innate immunity through interaction with cGAS-produced cyclic dinucleotides [150]. Human STING protein, consisting of 379 amino acids, has three domains: one N-terminal transmembrane helices (TM1–TM4) anchoring it to ER; one central cyclic di-GMP-binding domain (CBD) binding cyclic di-GMP for its activation; and one cytoplasmic C-terminal tail (CTT) activating TBK1 and IRF3 [151]. Stimulation by viral DNA induces conformational changes through binding DNA to cGAS, which activates enzymatic performance. cGAS can catalyze GTP and ATP transformation to cGAMP, the dinucleotide second messenger attaching onto ER-associated adaptor STING [152]. This prompts conformational changes and facilitates its trafficking from ER to Golgi apparatus via ER–Golgi intermediate compartment (ERGIC), in which it associates with and activates TBK1. The phosphorylated STING is a platform for IRF3 recruitment for phosphorylation via TBK1 [153]. The phosphorylated IRF3 dimerizes and moves into nucleus to enhance IFN-I transcription [153].

Research on autophagy-mediated regulation of cGAS/STING signaling in herpes simplex virus (HSV)-1 infection reveals an association of DNA-sensing with autophagy pathways. Beclin-1 is interactive with cGAS, inhibiting its nucleotidyl transferase activity, which subsequently reduces cGAMP production necessary for STING activation [154]. During human papillomavirus (HPV) infection, the cGAS–Beclin-1 interaction inhibits interferon production by reducing cGAMP synthesis and maintains systemic immune equilibrium by promoting autophagy-mediated cytosolic viral DNA degradation [154]. In DENV-infected HEK293T cells, autophagy targets cGAS, as a DENV NS2B protease cofactor binding cGAS, resulting in its lysosomal degradation and reduced type I IFN production, since cGAS cannot detect mitochondrial DNA released during infection [155]. In addition, cGAS undergoes ubiquitination via K63 linkage, which allows its recognition by the autophagy cargo receptor p62. This recognition promotes targeted autophagosomal degradation, thereby inhibiting cGAS–STING pathway [156]. However, Krause et al. demonstrated that the vaccinia virus (VACV) evades xenophagy through its interaction with SLRs. Specifically, VACV targets p62 in the initial stages of infection to prevent its degradation, suggesting that poxviruses modulate autophagic processes to promote cytoplasmic replication [157].

Continuous cGAS–STING pathway activation leads to STING degradation. This process has a key effect on keeping immune homeostasis [158]. ATG9SULK, the sole transmembrane protein of core autophagy mechanism, has negative regulation on dsDNA-induced immunity through inhibiting STING1 aggregation on compartments derived from Golgi apparatus, as well as STING and TBK1 assembly [159]. Moreover, cGAMP induces ULK1 dissociation from 5′-AMP-activated protein kinase catalytic subunit α2 (AMPK), thereby activating ULK1, which mediates the phosphorylation and degradation of STING. Activated STING triggers a nonclassical autophagy pathway that relies on ATG5 but does not depend on ULK1. Upon DNA stimulation, this autophagic mechanism either facilitates STING degradation or DNA clearance [160, 161]. In the later stages of HSV-1 infection, STING undergoes degradation via chaperone-mediated autophagy to attenuate IFN signaling [162]. According to Prabakaran et al., p62 is involved in mediating the active STING selective autophagy [163]. Specifically, p62 is responsible for recognizing K63-linked STING ubiquitination and targets it for autophagic degradation, thereby preventing the cGAS–STING pathway hyperactivation, which can result in subsequent IFN-I overproduction. Besides, as reported by Kimura et al., TRIM21 was also a selective autophagic receptor promoting IRF3 dimer autophagic degradation, so as to suppress IFN-I responses triggered by cGAS–STING pathway [137].

Many viruses can suppress STING activity. For example, PLP2-TM viral proteins in some coronaviruses (CoV), suxh as Middle East respiratory syndrome coronavirus (MERS-CoV) or SARS-CoV-1, promote the STING–Beclin-1 interaction. Such interaction generates the complex adversely regulating STING activity, finally decreasing IFN-I generation [164]. Moreover, nonstructural protein 6 (NSP6) in SARS-CoV-2 can induce ER stress-related autophagy to degrade STING and then reduce IFN generation [165, 166]. Furthermore, E7 protein of oncogenic HPV accelerates NLRX1-mediated autophagic STING degradation [167]. This is not related to proviral but to protumoral effect. To be specific, inhibiting STING-related IFN-I production can suppress T-cell infiltration in head and neck squamous cell carcinoma, facilitating immune evasion within tumors.

STING activation is found to promote noncanonical autophagy [168]. After STING is activated, it generates the highly conserved proton channel for deacidifying post-Golgi STING trafficking vesicles [169]. According to Xun et al., the proton channel exerted an essential effect on noncanonical activities of cGAS pathway, such as conjugating ATG8 lipidation on single membranes, called CASM [170]. Furthermore, STING trafficking is associated not only with TBK1 activation but also with the proton channel activity of STING. The channel is capable of initiating noncanonical LC3 lipidation, which involves CASMs, or V-ATPase-ATG16L1-mediated LC3 lipidation (VAIL) [171]. ATG8ylation, which refers to ATG8-family protein lipidation, is related to double-membrane autophagosome generation. Nonetheless, STING is linked to membrane ATG8ylation, noncanonical autophagy, and canonical autophagy processes, which are mostly associated with innate immunity and additional outcomes such as neuroinflammation [160, 161, 168]. Besides, ATG8ylation as LAP via tumor-associated macrophages exerts immunosuppression, as detected in the melanoma murine model, in which LAP suppresses STING-induced IFN-I response, a process necessary for infiltrating antitumor T-cell activation [172]. Such phenomenon is greatly significant, since the more potent STING-related IFN-I responses caused by inhibiting autophagy or ATG8ylation show benefits in tumor or tumor treatment models [173]. The precise functional role of CASM or ATG8ylation during STING signaling remains to be elucidated and will be of great interest for further understanding.

## Conclusions and perspectives

**Fig. 4:**
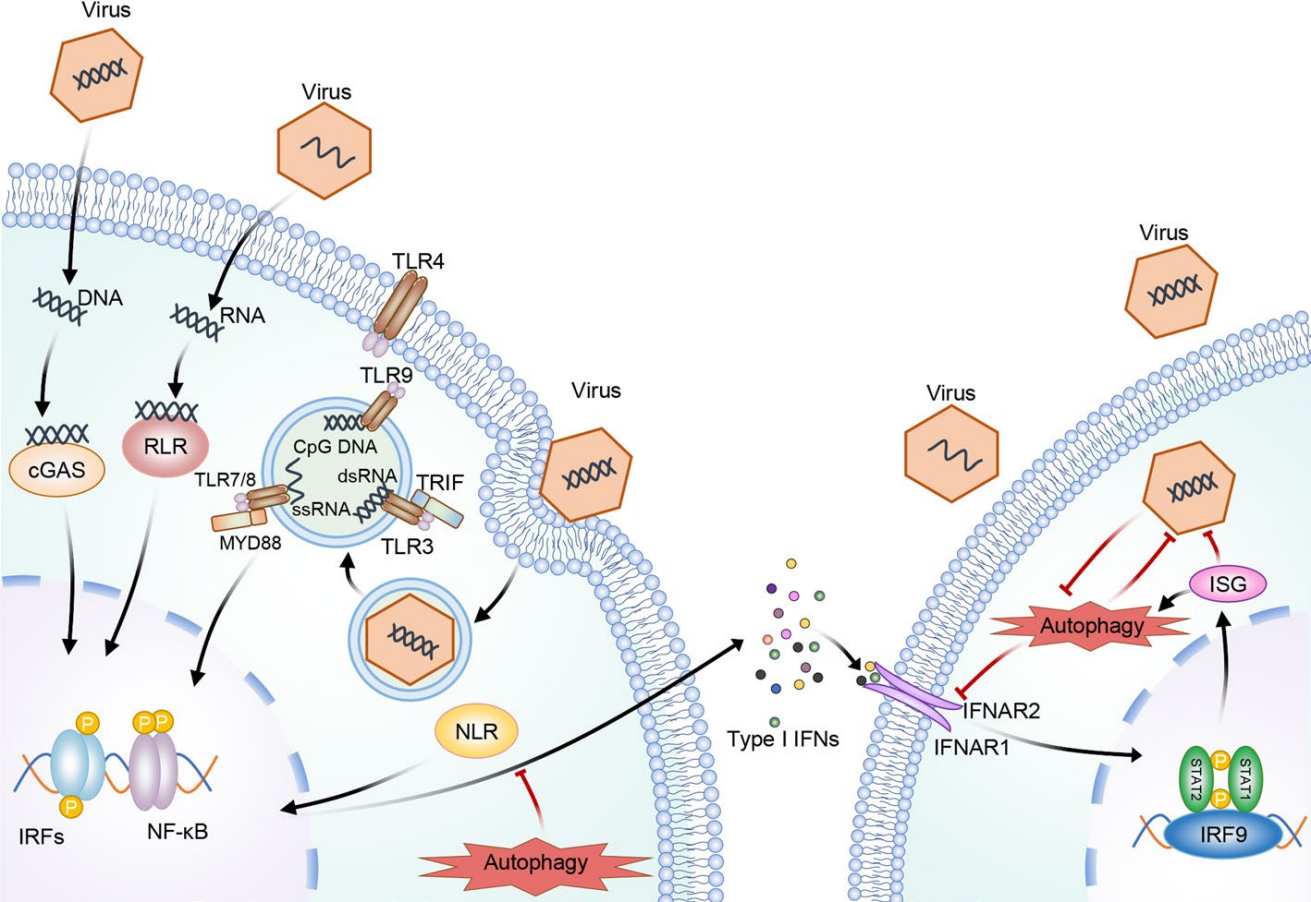
The crosstalk between autophagy and innate immunity in antiviral responses. Autophagy has a key function in innate immunity by interacting with IFN-I and PRRs pathways. Virus-induced autophagy has the capacity to suppress IFN-I antiviral responses, while the IFN-I system can modulate autophagy to facilitate viral clearance. The interaction between autophagy and PRRs potentially acts as a crucial link connecting autophagy to innate antiviral immunity. Solid arrows represent stimulation, whereas T-shaped arrows denote inhibition

Autophagy is the basic homeostatic mechanism in eukaryotic organisms, which has a key role in innate immunity by interacting with PRRs and IFN-I pathways (Fig. 4). It utilizes specific adaptors, particularly SLRs, for targeting and eradicating invading pathogens, such as viruses. Therefore, viruses evolve strategies for circumventing autophagic capture. During evolution, nearly all innate immunity components, such as traditional PRRs and inflammasomes, have developed complicated associations autophagy. In addition, such integration expands to adaptive immune system, notably affecting mammalian species.

Autophagic responses, which exert an essential effect on viral infections, are closely related to some virus families. Autophagy contributes to identifying and encapsulating viral components, and can limit viral replication and transmission through fusion with lysosomes for degradation, including herpesviruses. During HSV-1 infections, p62, the autophagy receptor, can target viral capsids for degradation [174, 175]. Likewise, in human cytomegalovirus (HCMV) infections, p62 can combine with viral proteins pp28 to degrade them and then reduce viral transmission [176]. On the contrary, if the autophagic mechanism is destroyed by viruses, it will promote viral replication to accelerate viral proliferation inside host cells and intensify infections. CoV, such as MERS-CoV [177], SARS-CoV [178], and SARS-CoV-2 [179, 180], can activate autophagy pathway, forming double-membrane vesicles (DMVs) to enhance viral replication. More research is warranted to illustrate the complicated autophagy–coronavirus interactions, which may help understand viral pathogenesis and develop innovative treatments. These progresses can greatly affect public health through providing more efficient approaches to prevent and treat viral infections.

The dual functions of autophagy in viral infections arouse wide attention recently. With regard to RNA viruses, such as murine norovirus (MNV), the convergence of ATG16L1 with TLR7 activation can enhance autophagy to limit viral replication [181]. In contrast, according to previous studies concerning enterovirus 71 (EV71), TLR7-driven autophagy promotes viral assembly [113]. Existing findings regarding the roles of viruses in inducing or inhibiting autophagy are contradictory, depending on the exact infection stage. For example, respiratory syncytial virus (RSV) can target Beclin-1 to suppress autophagosome maturation [182], while CoV enhances autophagy initiation and later suppresses the later stages for preventing viral component degradation [166, 183]. Such mechanisms suggest that there is complex interaction of autophagy modulation with viral pathogenesis. For certain viruses, enhancing autophagy can eliminate viral particles and alleviate inflammation. On the contrary, inhibiting autophagy avoids virus replication by using the above pathway. Therefore, such dual functions position autophagy as the candidate antiviral infection therapeutic target, probably alleviating the great public health burden.

The autophagy–immunity interaction in viral infection is a double-edged sword. On the one hand, PRR pathway activation after viral infection can trigger autophagy, thus promoting proinflammatory factor and IFN production for limiting viral replication. On the other hand, autophagy contributes to degrading impaired organelles and immune signaling proteins, thus probably impairing immunity. In extreme cases, it will prevent overactive immune responses and maintain intracellular homeostasis to promote viral replication. Therefore, deficiencies of autophagic mechanism components cause general immune, inflammatory, and autoimmune conditions within human bodies. In this regard, autophagy plays a key role in regulating viral replication and innate immunity. Table 1 presents different viral strategies for manipulating autophagy while mitigating antiviral innate immunity.

**Table 1:** Autophagy-regulated antiviral innate immunity manipulated by viruses

Virus	Autophagy manipulation mechanism	References
**RLR signaling pathway**		
HRV-16	Trehalose-mediated autophagy induces ATG5-MAVS-RIG-I interaction for reducing IFN-λ generation	[71]
HCV	Autophagy leads to ATG5 and Beclin-1 upregulation, which negatively affects RIG-I signal transduction	[73]
VSV	LRRC25 interacts with ISG15-modified RIG-I to facilitate interaction of RIG-I with autophagic cargo receptor p62	[74]
VSV	RNF34 is responsible for ubiquitinating MAVS, results in the NDP52-induced selective degradation through autophagy	[85]
HPIV3	Mitophagy degrades MAVS and interferes with RIG-I signal transduction-induced IFN-I responses	[77]
JEV	Inhibition of autophagy causes a rise in MAVS aggregation and strengthens innate responses	[78]
IAV	PB1-F2-mediated mitophagy promotes MAVS degradation for suppressing IFN-I generation	[79]
SARS-CoV-2	The ORF10 and M viral proteins facilitate mitophagy for autophagic degradation of MAVS	[80]
CSFV	NS5A viral protein facilitates a Beclin-1–MAVS interaction to reduce IFN-I production	[82]
CSFV	Autophagy suppresses the CSFV-mediated RIG-I–IRF3 and JAK-STAT signaling pathways to inhibit IFN-I generation	[83]
**TLR signaling pathway**		
HIV-1, SV5	Autophagy activates TLR7, which mediates antiviral cytokine production	[103, 104]
HCV	TLR7 and TLR8 recognize HCV genomic RNA and facilitate TNF-α generation	[105]
HCV	Autophagy depletes TRAF6, which limits host inflammatory cytokine production	[106]
EBV	Autophagic pathway activates TLR-mediated signaling in response in pDCs	[109]
IAV	Autophagy boosts H1N1 virus presence within lysosomes, triggering TLR signal transduction and aiding T-helper cell differentiation into BMDCs	[111]
EV71, CA16	Autophagy attenuated TLR7 activation	[113]
**NLRs/inflammasome signaling pathway**		
IAV	PB1-F2 viral protein is the autophagy receptor, promoting full mitochondrial autophagy through its interaction with LC3B and TUFM, resulting in MAVS degradation as well as decreased IFN-I generation	[79, 142]
IAV	PB1 viral protein targets MAVS to induce NBR1-relateda selective autophagic degradation	[144]
SARS-CoV-2	The accessory protein ORF10 promotes mitophagy-mediated MAVS degradation	[80]
**cGAS/STING signaling pathway**		
HSV-1	STING undergoes CMA-mediated degradation to terminate IFN signaling	[162]
HSV-1	p62 mediates the selective autophagy of active STING	[163]
HPV	The cGAS–Beclin-1 interaction curbs IFN production by lowering cGAMP synthesis and supports immune balance by enhancing autophagy to degrade cytosolic viral DNA	[154]
HPV	E7 viral protein promotes NLRX1-mediated STING autophagic degradation	[167]
DENV	NS2B protease cofactor binds cGAS and regulates the autophagic degradation	[155]
SARS-CoV-1, MERS-CoV	PLP2-TM viral proteins facilitate the interaction between STING and Beclin-1	[164]
SARS-CoV-2	The NSP6 viral proteins induces ER stress-mediated autophagy, resulting in STING degradation and a subsequent decrease in IFN production	[165]

cGAMP, cyclic GMP–AMP; cGAS, cyclic GMP–AMP synthase; CMA, chaperone-mediated autophagy; CSFV, classical swine fever virus; DENV, Dengue virus; ER, endoplasmic reticulum; HCV, hepatitis C virus; HPV, human papillomavirus; HSV, herpes simplex virus; IAV, influenza A virus; IFN, interferon; IRF3, interferon regulatory factor 3; ISG, interferon-stimulated gene; JEV, Japanese encephalitis virus; LC3, protein L chain 3; MAVS, mitochondrial antiviral-signaling; NF-κB, nuclear factor kappa B; NLRs, nod-like receptors; NLRPs, Nucleotide-binding oligomerization domain, leucine-rich repeat and pyrin domain-containings; RIG-I, retinoic-acid-inducible gene I; RLRs, retinoic-acid-inducible reportors; SARS-CoV-2, severe acute respiratory syndrome coronavirus; TLRs, Toll-like receptors; TNF-α, tumor necrosis factor-alpha; VSV, vesicular stomatitis virus

Innate immune responses have been extensively suggested to cause harm through the precipitation uncontrolled inflammation in viral infections, causing serious clinical manifestations or even death. Such phenomenon is exemplified by SARS-CoV-2 infections that induce COVID-19 pandemic. In the COVID-19 pandemic, autophagy is an important process for modulating viral replication and innate immunity. As autophagy has important effects, comprehensive analysis is needed to explore effects of autophagy-regulating pharmacological agents on viral infections. Therefore, it is urgently needed to comprehensively and thoroughly investigate interactions of viral infections, host immunity, and treatments. The present work suggests that autophagy exerts an essential effect on host–pathogen interactions, especially in the antiviral infection immunity and exploitation by viruses. Therefore, an advanced understanding of autophagy in this aspect to antiviral innate immunity is essential for creating new treatments that address viral replication and immunity against infections in the host.

## Data Availability

No datasets were generated or analyzed during the current study.
